# The Usefulness of Radiomics Methodology for Developing Descriptive and Prognostic Image-Based Phenotyping in the Aging Population: Results From a Small Feasibility Study

**DOI:** 10.3389/fragi.2022.853671

**Published:** 2022-04-28

**Authors:** Rebeca Mirón Mombiela, Consuelo Borrás

**Affiliations:** ^1^ Herlev og Gentofte Hospital, Herlev, Denmark; ^2^ Freshage Research Group, Department of Physiology, Faculty of Medicine, Institute of Health Research-INCLIVA, University of Valencia, and CIBERFES, Valencia, Spain

**Keywords:** frailty, radiomics, mitocondrial, phenotyping, muscle, ultrasound

## Abstract

**Background:** Radiomics is an emerging field that translates medical images into quantitative data to enable phenotypic profiling of human disease. In this retrospective study, we asked whether it is possible to use image-based phenotyping to describe and determine prognostic factors in the aging population.

**Methods:** A radiomic frailty cohort with 101 patients was included in the analysis (65 ± 15 years, 55 men). A total of 44 texture features were extracted from the segmented muscle area of the ultrasound images of the anterior thigh. Univariate and multivariate analyses were performed to assess the image data sets and clinical data.

**Results:** Our results showed that the heterogeneity of muscle was associated with an increased incidence of hearing impairment, stroke, myocardial infarction, dementia/memory loss, and falls in the following two years. Regression analysis revealed a muscle radiomic model with 87.1% correct predictive value with good sensitivity and moderate specificity (*p* = 0.001).

**Conclusion:** It is possible to develop and identify image-based phenotypes in the elderly population. The muscle radiomic model needs to further be validated. Future studies correlated with biological data (genomics, transcriptomics, metabolomics, etc.) will give further insights into the biological basis and molecular processes of the developed radiomic model.

## Introduction

Medical imaging provides the ability to detect and localize many anatomical or physiological changes that are important to determine whether a disease is present or if therapy is effective (Prescott, 2013), but to date, we describe complex diseases with simple numbers, such as the thickness or cross-sectional area of your muscle. Current markers for Frailty are neither sensitive nor specific enough to identify differences in disease phenotype and might inaccurately suggest a similarity in the contribution of various frailty risk factors or adverse events to disease progression (Alvarez-Bustos et al., 2021).

The field of radiology is going through a revolution, starting with an increased number of pattern recognition tools. These advances have facilitated the development of “Radiomics”, a precision tool that extracts intensity, shape, size, or texture from medical images using data-characterization algorithms (Tomaszewski and Gillies, 2021). This methodology has the power to translate medical images into quantitative data to enable phenotypic profiling of human disease. It is motivated by the concept that biomedical images contain information that reflects underlying pathophysiology, and these relationships can be revealed via quantitative image analysis that offers information on the disease microenvironment and the disease state. This data is then used to build diagnostic or predictive phenotypes. It has mostly been used in the field of oncology for treatment response, outcome prediction, and assessment of cancer genetics, otherwise known as tumor phenotyping. (Gillies et al., 2016).

Our research group has focused on researching and developing quantitative imaging biomarkers for the diagnosis, treatment, follow-up, and risk assessment of frailty. In this Brief Research Report, we would like to highlight the possible application of Radiomics as a research method in the aging population. We believe it can also be used to take a fresh look at other pathophysiological processes, such as frailty. In this retrospective study, we asked whether it is possible to use image-based phenotyping to describe and determine prognostic factors in the aging population. We will also explore and explain the usefulness and applicability of such methodology, as well as its current limitations.

## Methods

### Study Design

**FIGURE 1:**
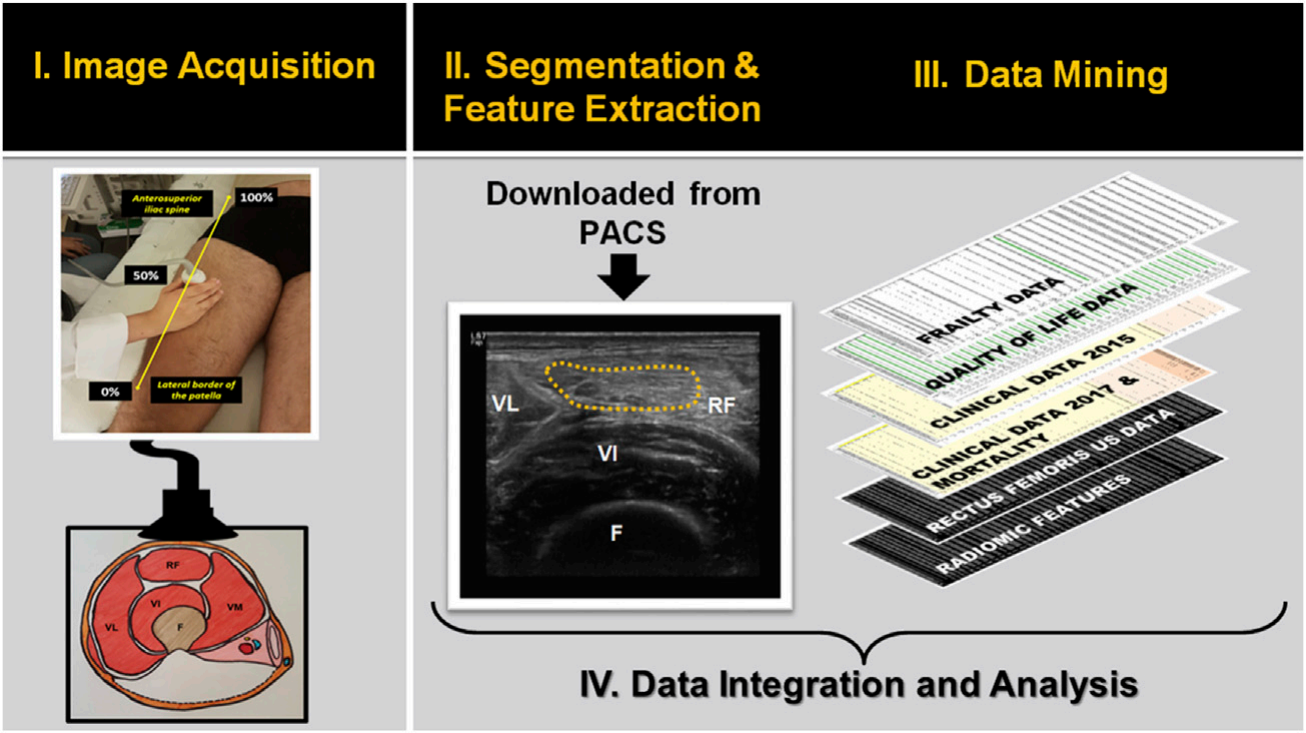
Flowchart showing the main steps for developing descriptive and prognostic image-based phenotyping using radiomic features extracted from ultrasound images. Abbreviations: MT: muscle thickness; VL: Vastus lateralis; RF: rectus femoris muscle; VI: vastus intermedius; F: Femur; PACS: Picture archiving and communication system.

This was an exploratory study of previously acquired ultrasound data (November 2014—February 2015) and its corresponding follow-up clinical data (March 2017). The cohort consisted of 112 patients between 20 and 90 years old that previously participated in a cross-sectional frailty study (64 ± 15 years, 61 men) (Mirón Mombiela et al., 2017). We only excluded participants if image data was missing from the original study. Radiomic features were extracted from the segmented muscle area of the ultrasound images of the anterior thigh. The clinical data mining included obtaining physical characteristics, muscle performance data, frailty phenotype, and quality of life. We also recorded about 30 comorbidities and risk factors from the patient’s medical history at baseline and its incidence two years after the ultrasound scanning. Univariate and multivariate analyses were performed to assess associations of the radiomics features with the clinical data (Figure 1). All research was carried out by the relevant Spanish legislation and adhered to the principles of bioethics included in the Declaration of Helsinki. The Institutional Research and Ethics Committees approved the study, and each patient gave written informed consent before entering the original study.

### Radiomics Features

We defined 44 radiomic image features that are used to describe and characterize tissues and can be extracted in an automated way. Four features were extracted from the intensity histogram (first-order statistics) and the other 40 features were extracted from second-order and higher-order statistical methods: from the gray-level co-occurrence matrix (GLCM) 9 features were derived, from the gray-level run-length matrix (GLRLM) 13 features, from the gray-level size zone matrix (GLSZM) 13 features and from the neighborhood gray-tone difference matrix (NGTDM) only 5. The features were extracted using the MATLAB toolbox Radiomics implemented by Vallières and others (Vallieres et al., 2015). The full list of the radiomic features used in this study can be found in Supplementary Table S1.

### Image Data Set

We applied a radiomic analysis to one previously acquired image data set (Mirón Mombiela et al., 2017). The data set consisted of 7 ultrasound images from 101 patients after excluding those with incomplete image data. The measured site was the midpoint between the superior border of the patella and the anterior superior iliac spine, with the transducer positioned perpendicular to the longitudinal axis of the femoral quadriceps (Mirón Mombiela et al., 2021). Three cross-sectional images contained annotations from the previous study and one longitudinal image were excluded from the analysis. The other 3 cross-sectional images, all corresponding to the anterior compartment of the right thigh, were downloaded from the Picture Archiving and Communication System (PACS) of the hospital, to perform the data segmentation and feature extraction. The data segmentation was performed by a radiologist with three years-experience in muscle ultrasound (R.M.M.). Each muscle region of interest (ROI) was manually segmented in 2D from the cross-sectional images of the *rectus femoris* muscle. The segmented ROI excluded bone, fatty tissue, muscle fascia, and the internal tendon of the *rectus femoris* muscle.

### Clinical Data Set

At the time of the ultrasound scanning the following information was collected: 1) physical characteristics that included patients age, sex, weight, height, and body mass index (BMI); 2) muscle performance that included muscle strength and gait speed; 3) the Frailty Criteria (Fried et al., 2001); 4) quality of life that was based on the use of the generic questionnaire for the elderly, known as OPQOL-35 (Older’s People Quality of Life) (Bowling, 2009); and 5) comorbidities and risk factors. We recorded the presence or absence of 30 comorbidities and risk factors and we followed up its incidence for two years. We included hypertension, hyperlipidemia, diabetes mellitus, chronic obstructive pulmonary disease (COPD), hearing or visual impairment developed in the last 6 months, Parkinson’s disease, stroke, congestive heart failure, heart disease (that is not heart failure nor myocardial infarction), myocardial infarction, renal disease, previous cancer, arthritis and/or osteoarthritis, anxiety syndrome, depression, previous fractures or osteoporosis, liver disease or hepatopathy, dementia or memory loss, connective tissue disease, hemiplegia, neoplasm, leukemia, malignant lymphoma, solid metastasis, acquired-immunodeficiency syndrome (AIDS), and peripheral vascular disease. The risk factors included smoking, alcohol consumption, falls, and obesity. Finally, we recorded the number of visits to a primary care physician, to the emergency department, and hospital admissions in the last six months. Two years after the ultrasound examination, we reviewed the subject’s medical records and did a follow-up on the same variables described before, and recorded its incidence. The full description of how the frailty phenotype was obtained, including grip strength and gait speed, and the classification into groups can be found in Supplementary Appendix A1.

### Sample Size

In texture analysis, the discriminative power of the predictive model is dependent on having sufficient data. Radiomic analysis can be performed with as few as 100 patients (Gillies et al., 2016). Our study sample was calculated to meet this criterion. No other sample calculations were performed.

### Data Analysis

Descriptive data are presented with mean ± standard deviation (SD) with the distribution of the data verified by the Kolmogorov-Smirnov normality test. The evaluations of the different variables of the study according to frailty phenotype and control group were determined using ANOVA for the parametric variables, and the non-parametric Kruskal-Wallis test was used. Correlations were performed to investigate the relationship between physical characteristics, muscle performance data, frailty phenotype, quality of life, the incidence of comorbidities, and/or risk factors, with the radiomic features. We used the coefficient of Pearson (r) for parametric data, Spearman’s Rank for non-parametric data, and Tau B of Kendal used for ordinal variables. Heatmaps and Manhattan plots were used to graphically represent the data and to better visualize the value of the associations within the data. Hierarchical and K-Mean cluster analysis was performed to identify the most relevant grouping of radiomic features for the radiomic signature. Once a radiomic phenotype and corresponding features were identified a multiple logistic regression analysis was performed. We applied a false discovery method by Benjamini and Hochberg as multiple comparisons were performed during the study. All statistical analyses were performed with SPSS version 24.0 for Windows (IBM SPSS, Inc., Chicago, IL).

## Results

The sample was composed of 101 patients (64 ± 15 years, 55 men) and there were 24 controls, 22 robust, 30 pre-frail, and 25 frail patients (Supplementary Table S2). The physical characteristics (age, weight, height, BMI, gait speed, and muscle strength), ultrasound measurements (muscle thickness and subcutaneous fat thickness), quality of life, sex, and frailty criteria distributed by the frailty phenotype and the control group are shown in Supplementary Table S2. The data shows that there are statistically significant differences between age, height, BMI, gait speed, muscle thickness, and quality of life, depending on the frailty phenotype. The weight and subcutaneous fat thickness were distributed homogeneously within the groups. In addition, females and males were also homogeneously distributed. Supplementary Table S2 also shows the number of positive criteria subjects had in each group, explaining how patients were classified in the study. For comparison reasons, we also applied the frailty criteria to controls.

### Comorbidities at Baseline and Follow-Up

The analysis of comorbidities according to frailty phenotype (Supplementary Table S3), shows how the distribution changes among groups and increases in frequency at follow-up. At baseline, 11 out of 34 comorbidities surveyed were heterogeneous between the groups, and at follow-up 19 out of 35. Among the comorbidities that were statistically significant according to frailty phenotype at baseline and follow-up are hypertension, hyperlipidemia, diabetes mellitus, visual impairment, stroke, congestive heart failure, heart disease, renal disease, previous cancer, arthritis/osteoarthritis, anxiety/depression, falls, obesity, number of visits to primary care, number of visits to the emergency department, and hospitalizations. There was no change in the prevalence of new cases of leukemia, AIDS, Lymphoma, and alcohol consumption, so variables were excluded from further analysis.

### Association of Radiomic Data With Clinical Data

We used multiple correlations of all the variables in this study with the purpose to perform data mining, which refers to the process of discovering patterns in large data sets (Gillies et al., 2016) associated with imaging for further analysis. Radiomic features had statistically significant correlations with age, gait speed, frailty phenotype, subcutaneous fat tissue, and muscle thickness (Supplementary Figure S1). Weight, height, BMI, muscle strength, and quality of life had mostly weak to moderate associations with a few to no significant correlations with the radiomic features.

### Prognostic Value of Radiomic Data

**FIGURE 2:**
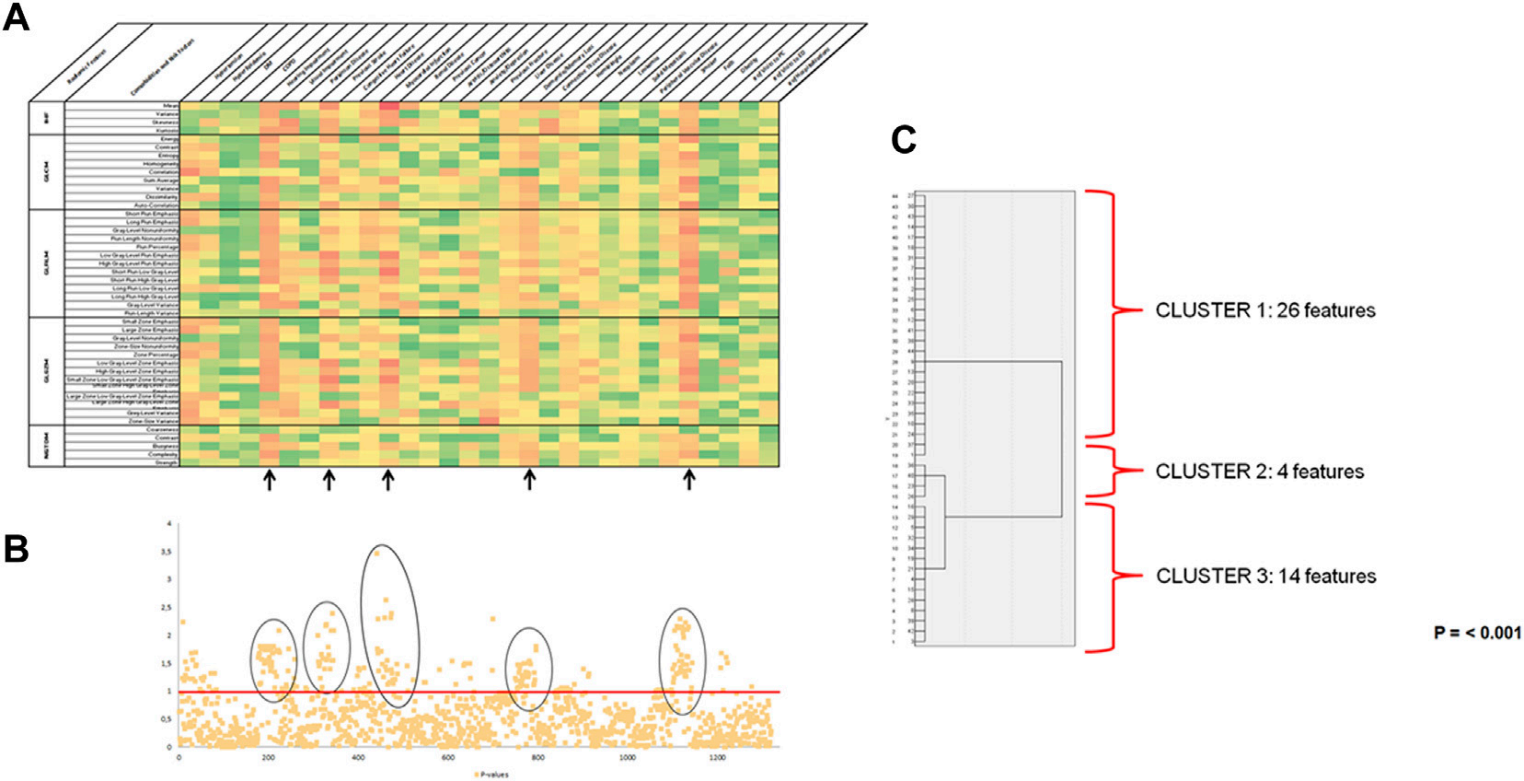
Prognostic value of radiomic data. **(A)** Heatmap of Rho of Spearman Correlation coefficients for an association of Radiomic Features and Incidence of new diseases and risk factors (*n* = 101). On the *x*-axis, radiomics features are shown, and on the *y*-axis are the incidence of comorbidities and risk factors. The elements of the heatmap are color-coded depending on the value of the correlation coefficient. Red is for the highest value and green for the lowest, with 5 different colors in between. Abbreviations: DM: Diabetes Mellitus; COPD: Chronic Obstructive Pulmonary Disease; #: number; PC: primary care; ED: emergency department; IHF: Intensity Histogram Features; GLCM: Gray-Level Co-occurrence Matrix; GLRLM: Gray-Level run-Length Matrix; GLSZM: Gray-Level Size Zone Matrix; NGTDM: Neighborhood Gray-Tone Difference Matrix. Note. Tau B of Kendal was used for the statistical analysis. **(B)** Manhattan plot of *p*-values for associations between radiomic features and incidence of new diseases and risk factors (*n* = 101). *p*-values for univariate associations between each radiomic feature and the incidence of new disease and risk factors after 2 years of following from baseline ultrasound. Radiomic features are situated on the *x*-axis in the same order as the heatmap, while the corresponding *p*-values are located on the *y*-axis and graph with a -LOG10 (*p*-value) scale. Points above the red line (*p*= <0.05) indicate radiomic features in which case the incidence of new diseases or risk factors showed significant association. **(C)** Hierarchical cluster dendrogram (*n* = 44). Hierarchical cluster dendrogram of radiomic features significantly associated with hearing impairment, stroke, myocardial infarction, dementia or memory loss, and falls. Three independent clusters are identified for the radiomic phenotype (*p* = 0.001).

Both the Heatmap and Manhattan-Plot for radiomic features and incidence of new diseases and risk factors showed weak to moderate statistically significant associations (Figures 2A,B) between the radiomic features taken at baseline and the development of hearing impairment (35 out of 44 features), stroke (37 out of 44 features), myocardial infarction (37 out of 44 features), dementia/memory loss (30 out of 44 features) and falls (29 out of 44 features) after 2 years of follow-up. All other variables evaluated lacked statistically significant associations or the association was weak and were excluded from any further analysis (like for example hypertension and number of visits to primary care). These four diseases and one risk factor were identified as radiomic signature or phenotype, and their correlation coefficient values were further evaluated to identify the radiomic features that could be used for constructing a predictive model. Hierarchical and K-mean cluster analysis was used for this purpose (Figure 2C) and identified three independent clusters of radiomic features that were statistically significant (*p* = 0.001). The complete list of radiomic features corresponding to each cluster is found in the supplemental materials (Supplementary Table S4).

### Prognostic Validation of Radiomic Phenotype

The muscle radiomic signature or phenotype identified a total of 22 subjects that developed 29 newly diagnosed comorbidities or events (2 hearing impairments, 4 strokes, 7 myocardial infarctions, 11 dementia/memory loss, and 5 falls) in the following two years. For the full characteristics of the sample according to identified muscle radiomic phenotype see Supplementary Table S4. To quantify the effects of each radiomic predictive model on the likelihood of developing hearing impairment, stroke, myocardial infarction, dementia/memory loss, and/or falls, a multiple logistic regression analysis was applied to the identified radiomic feature clusters. The logistic regression analysis for all three models (Table 1) was statistically significant (*p* = 0.029 to 0.001). The models explained 34.80–63% (Nagelkerke R2) of the variance in the radiomic models. Although the data showed correct predictions ranging from 78.1 to 87.1%, the regression analysis plots revealed that only Model 1 was predictive of the outcome with good sensitivity and moderate specificity. The other two models were good at excluding disease or identifying individuals without this radiomic phenotype. This is corroborated by the high sensitivity and low specificity of both models.

**TABLE 1:** Multivariate Logistic Regression Analysis of Muscle Radiomic Phenotype Predictive Models (events = 29, *n* = 101).

Radiomic Signature Models	Model Chi-Square [df]	% Correct Predictions	Sensitivity	Specificity	Hosmer and Lemeshow Test	Nagelkerke-R2	Plot
Radiomic Model 1	53.54 [25], *p* = 0.001	87.1	92.4	68.2	0.999	0.634	Predictive
Radiomic Model 2	10.75 [4], *p* = 0.029	78.2	97.5	9.1	0.699	0.348	Not Predictive
Radiomic Model 3	32.43 [14], *p* = 0.003	82.2	93.7	40.9	407	0.407	Not Predictive

### Stepwise Logistic Regression Analysis of Radiomic Model 1

A stepwise logistic regression analysis was applied to the best model (see Table 2) to test the relation with the other relevant clinical data identified (age, gait speed, frailty phenotype, subcutaneous fat tissue, and muscle thickness) that were significant to the muscle radiomic phenotype (Supplementary Table S5) and the radiomics features individually (Supplementary Figure S1). Block one shows in detail the relation between the muscle radiomic phenotype (dependent variable) and the radiomics features that constituted model 1. Short Run Emphasis of GLRLM, Run Length Nonuniformity of GLRLM, Variance of pixels, Contrast of GLCM, Grey-Level Variance of GLSZM, Strength of NGTDM, Long Run High Gray-Level of GLRLM, and Large Zone High Gray-Level Zone Emphasis of GLSZM were statistically significant (*p* < 0.05) and had the strongest explanatory weight on the model. The second block assessed the value of the strong correlated clinical variables with the radiomic model 1, but because of the high amount of variables included, all variables lose statistical significance. For this reason, we recalibrate the model excluding the radiomic feature with a Wald Statistic close to cero or cero and evaluated Block 3 with the clinical variables again. Muscle thickness, subcutaneous fat thickness, age, and gait speed had a strong explanatory weight with the model, but no statistical significance. The frailty phenotype had neither. This could be due to the suppression effect due to the close nature between the variables (age, frailty phenotype, and gait speed). Therefore, block 4 evaluated the effect of only age and frailty phenotype on the model given the big range of ages in the sample. Frailty phenotype had a higher weight with the model and was statistically significant, yet age was not statistically significant to the model. And although blocks 3 and 4 were statistically significant to the overall model, they did not improve substantially the predictive capacity of the models.

**TABLE 2:** Stepwise multivariate logistic regression analysis of radiomic model 1.

Variables	Radiomic features that constitute the radiomic model 1		Adjustment after strong correlated variables		Calibration of the radiomic model		Effect of age and frailty phenotype on the radiomic model	
	Wald statistic*	*p*-value	Wald statistic*	*p*-value	Wald statistic*	*p*-value	Wald statistic*	*p*-value
Radiomic signature (dependent variable)	4,703	0.030	0.021	0.884	6,241	0.012	3,181	0.075
Small Zone Emphasis of GLSZM	2,162	0.141	0.018	0.892	2,407	0.121	0.553	0.457
Zone-Size Nonuniformity of GLSZM	2,758	0.097	0.018	0.892	2,875	0.090	1,030	0.310
Complexity of NGTDM	1,201	0.273	0.010	0.922	1,411	0.235	1,345	0.246
Short Run Emphasis of GLRLM	4,563	0.033	0.021	0.884	5,607	0.018	2,444	0.118
Run Length Nonuniformity of GLRLM	4,707	0.030	0.022	0.881	5,866	0.015	2,834	0.092
Run Percentage of GLRLM	0.992	0.319	0.019	0.891	1,276	0.259	0.107	0.744
Zone Percentage of GLSZM	2,851	0.091	0.021	0.886	2,739	0.098	2,461	0.117
Entropy of GLCM	1,033	0.309	0.000	0.999			0.008	0.930
Variance of GLCM	3,568	0.059	0.022	0.881	5,141	0.023	4,270	0.039
Variance of pixels	4,049	0.044	0.004	0.948	1,118	0.290	4,334	0.037
Gray-Level Variance of GLRLM	0.287	0.592	0.002	0.966	2,225	0.136	0.516	0.472
Contrast of GLCM	4,101	0.043	0.016	0.898	4,535	0.033	2,165	0.141
Dissimilarity of GLCM	2,920	0.087	0.018	0.894	2,225	0.136	0.001	0.970
Contrast of NGTDM	0.757	0.384	0.021	0.885	1,118	0.290	3,962	0.047
Grey-Level Variance of GLSZM	4,237	0.040	0.002	0.962			1,283	0.257
Strength of NGTDM	4,513	0.034	0.001	0.980			0.985	0.321
Correlation of GLCM	1,229	0.268	0.001	0.974			0.024	0.878
Auto-Correlation of GLCM	3,342	0.068	0.001	0.978			0.661	0.416
High Gray-Level Run Emphasis of GLRLM	2,369	0.124	0.016	0.900	3,780	0.052	0.808	0.369
Short Run High Gray-Level of GLRLM	2,989	0.084	0.016	0.898	4,735	0.030	2,570	0.109
High Gray-Level Zone Emphasis of GLSZM	0.283	0.595	0.002	0.969			0.657	0.417
Small Zone High Gray-Level Zone Emphasis of GLSZM	1,575	0.209	0.016	0.900	5,125	0.024	2,626	0.105
Long Run High Gray-Level of GLRLM	4,335	0.037	0.023	0.881	3,323	0.068	4,388	0.036
Large Zone High Gray-Level Zone Emphasis of GLSZM	4,352	0.037	0.023	0.878	5,314	0.021	4,461	0.035
Mean of pixels	1,902	0.168	0.014	0.905	2,954	0.086	1,997	0.158
Muscle Thickness (cm)			0.005	0.943	1,839	0.175		
Subcutaneous Fat Thickness (cm)			0.015	0.902	2,660	0.103		
Age (years)			0.022	0.883	3,552	0.059	2,282	0.131
Frailty Phenotype (Non-Frail/Frail)			0.000	0.990	0.012	0.914	3,846	0.050
Muscle Strength (kg)			0.003	0.954				
Gait Speed (s)			0.013	0.910	2,207	0.137		
Block Chi-Square [df]	-		53.67 [6], *p* = <0.001		24.52 [5], *p* = 0,<0.001		15.86 [2], *p* = <0.001	
Model Chi-Square [df]	52.21 [25], *p* = 0.001		105.88 [30], *p* = <0.001		61.31 [22], *p* = <0.001		69.40 [27], *p* = <0.001	
Nagelkerke-R2	0.621		1,000		0.777		0.765	
Hosmer y Lemeshow Test	0.069		1,000		0.970		0.922	
Correct Predictions (%)	87.1		100.0		90.1		89.1	

Abbreviations: GLCM, Gray-Level Co-occurrence Matrix; GLRLM, Gray-Level run-Length Matrix; GLSZM, Gray-Level Size Zone Matrix; NGTDM, Neighborhood gray-tone difference matrix. *The Wald statistics are distributed chi-square with 1 degree of freedom. Sum Average of GLCM, was excluded of all statistical analysis because a Wald statistic equal to cero.

### False Discovery Rate for Multiple Comparisons

This study tested the significance of thousands of variables, which creates serious concerns over the accumulated Type 1 error. Many of the significant developments within the field of so-called “large-p, small-n” data analysis problems are robust methods for the accommodation of multiple testing issues (Kumar et al., 2012). We applied the FDR method to give reasonable guidance on the validity of our results (Benjamini and Cohen, 2017). The q-value in this study is 4.6%, which means that approximately less than 5% of significant results are false positives.

## Discussion

Radiomics aims to capture the informative content hidden in medical images, overcoming the limitations of the human eyes and human cognitive patterns (Gatta et al., 2020). These patterns can be expressed in terms of macroscopic image-based radiomic features. This allows us to infer phenotypes or signatures, possibly containing prognostic information from quantitative analysis of routine medical image data (Lambin et al., 2012).

**FIGURE 3:**
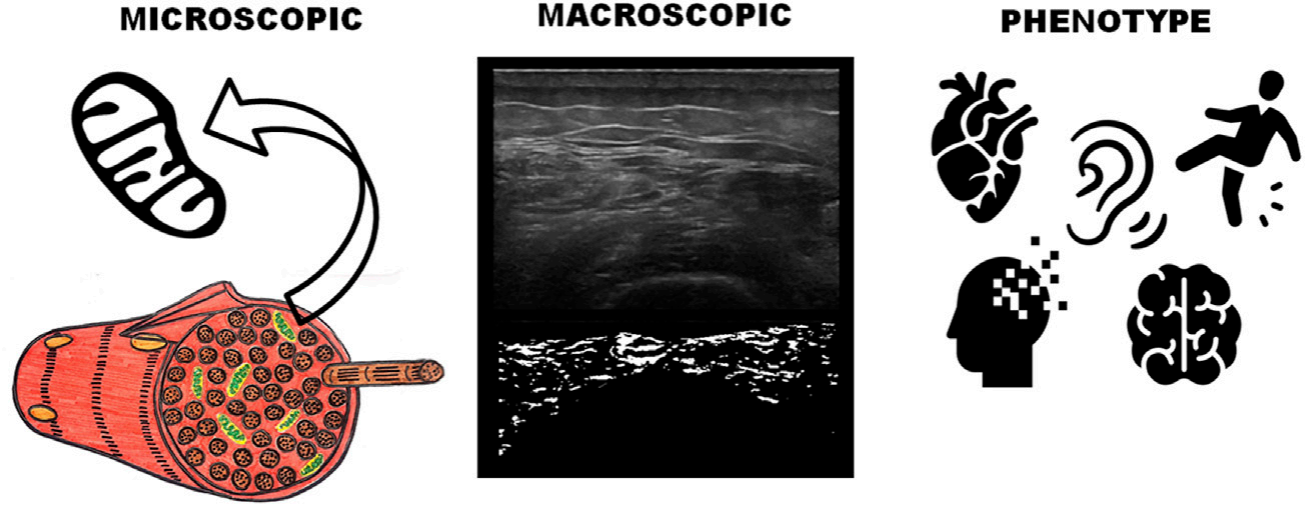
Mitochondrial radiomic signature of ultrasound images. Radiomics aims to capture the informative content hidden in medical images, overcoming the limitations of the human eyes and human cognitive patterns. These patterns can be expressed in terms of macroscopic image-based radiomic features and carry information about their underlying pathophysiological processes and pinpoint specific biological mechanisms. This allows us to infer phenotypes or signatures, including prognostic information. Here we graphically showed that a radiomic phenotype, capturing the muscle heterogeneity, was strongly prognostic of the development of hearing impairment, stroke, myocardial infarction, dementia/memory loss, and/or falls. Based on the type of disease associated with the muscle ultrasound changes, we also believe this identified group of diseases shares a mitochondrial link. Icons utilized in this figure were obtain from the Noun Project from the following authors: Gorkem Oner (mitochondria), Gregor Cresnar (ear), Artem Kovyazin (brain), Tatina Vazest (heart), Luis Padra (fading head) and Visual Language Company (slipping person).

This study shows that it is possible to use image-based phenotyping or radiomics, to describe and determine prognostic factors in the aging population (Figure 3). First, the image analysis revealed a distinct radiomic phenotype/signature capturing an association between muscle heterogeneity detected by ultrasound radiomic features and the incidence of hearing impairment, stroke, myocardial infarction, dementia/memory loss, and falls in the following two years (Figure 2 and Supplementary Table S4). Second, our sample did develop a variety of comorbidities in the following two years after the ultrasound scanning (Supplementary Table S3), but the radiomic features extracted from muscle seem to be insensitive to all other newly diagnosed comorbidities (Figure 2A). Thus establishing a relationship between the radiomic features extracted from muscle ultrasound and hearing impairment, stroke, myocardial infarction, dementia/memory loss, and falls found in this sample. Lastly, the regression analysis provides evidence of a good statistically significant predictive model (Table 1), allowing us to imply the prognostic value of muscle ultrasound scanning. Even after being adjusted for several strong correlated variables, the model remains stable where the adjusted variables had little effect on the prediction capacity of the model (Table 2).

The prognostic model we propose is simple and can be built solely from data extracted from a routine muscle ultrasound examination. Radiomic signature consisted of 26 radiomic features, 4 focused on the variance of the pixels, 8 focused on the distribution of pixels with high gray-level, and 14 focused on the amount of heterogeneity the distribution of the pixels had. Not all features were evenly weighted; as some had higher Walt statistics concerning the model (Table 2). In biological terms, the individual components of the radiomic model describe patients that develop the radiomic phenotype. Theses patients had an altered muscle structure, characterized with increased muscle echo intensity or higher grey-levels and heterogeneity or patchy distribution of those pixels with high grey-levels in the ultrasound images. While the other two models that were good at excluding the radiomics phenotype where characterized by radiomics features that focused on the distribution or presence of low grey-levels and homogeneity (Supplementary Table S4), imaging characteristics of a normal-looking muscle. Muscle quality, expressed as echo-intensity, has been researched with great interest and ultrasound can identify structural changes in the muscle caused by muscle degeneration, specifically the increase of adipose and intramuscular connective tissue that results in an increased echo-intensity of the assessed muscle (Pillen et al., 2009; Wilhelm et al., 2014; Akima et al., 2016). Studies that have used fractal dimension, a texture radiomic feature, to characterize muscle changes with ultrasound, have found similar evidence of increased connective tissue and fiber disorganization in the muscle as echo-intensity and heterogeneity increases (Cury et al., 2018; Mirón Mombiela et al., 2021).

When aspects of the mitochondrial theory of aging were examined in single muscle fibers instead of whole muscle homogenates, it was discovered that some segments of fibers were completely deficient for electron transport chain (ETC) activity. There was a near-complete absence of normal mtDNA in these regions, suggesting that ETC activity was closely linked to the status of mtDNA in the region. Furthermore, fibers displaying these mtDNA deletions and ETC deficiencies were also significantly atrophied. These observations suggest that mtDNA deletions may appear in a stochastic manner in small segments of single fibers leading to the death of a segment of a fiber (Johnston et al., 2008). These findings may explain why one of the most predominant findings of muscle ultrasound in this study in the aging population is increased heterogeneity measured by its echo intensity.

Given the range of different ages (range from 20 to 90 years old) included in the study, one might question the strong predictive power of radiomics phenotype is because of the number of younger patients included in the control and robust group. Others might also think as odd to include a control group in a non-experimental design, but the presence of control groups allows researchers to confirm that study results are due to either manipulation of independent variables or inherent characteristics of the patients being studied rather than extraneous variables. For this reason, we conducted an analysis of the age and frailty phenotype effect had on the radiomic model (Table 2). The results show that age was not statistically significant and had a small effect on the model, reducing the possibility of confirmation bias due to the ages included in the sample. On the other hand, frailty Wald statistics and *p*-value change erratically in response to small changes in the model from one block to the other one. This could be because of multicollinearity of the variable, as the Fried frailty phenotype involves muscle dysfunction at its core and increased echo-intensity has been reported associated with muscle strength, muscle thickness, and gait speed—markers with strong relationships with frailty status in previous research (Bartley and Studenski, 2017). Ultimately, multicollinearity does not reduce the predictive power or reliability of the model as a whole, it only affects calculations regarding individual predictors.

These results thus lend further credence to earlier studies. One is that radiological images can detect structural muscle changes in the aging population and that these changes can predict important clinical outcomes, implying the importance of detecting muscle quality in radiological images (Boutin and Lenchik, 2020; Pickhardt et al., 2021). Notwithstanding, it also adds to the current body of literature the capacity of medical images, in this case, muscle ultrasound, to predict adverse outcomes and identify those at risk of serious disease and substantial disability. The ultrasound muscle changes in the aging subjects that we report here are also reminiscent of other published evidence that indicate that ultrasound assessment of muscle can predict the length of stay in the intensive care unit (Gruther et al., 2008), postsurgical complications (Mueller et al., 2016), frailty (Shah et al., 2019), decreased quality of life (Mirón Mombiela et al., 2017), and/or survival (Greening et al., 2015; Lee et al., 2021; Yanagi et al., 2021). We build on that statement that muscle changes can predict the new onset of hearing impairment, stroke, myocardial infarction, dementia/memory loss, and/or falls in a subgroup of aging individuals using radiomics methodology.

### Usefulness and Applicability of Radiomic Methodology

One of the main benefits is that medical imaging data acquired in routine healthcare can be used in a new way to inform clinicians about the biology of a disease and provide potential prognostic or predictive information (O'Connor et al., 2015). With a rather wide variability in the prognosis of aging individuals, there is an urgent need for more precise and readily define prognostic parameters to group patients according to disease risk, to facilitate treatment options, and radiomics methodology could provide that.

### Strengths and Limitations

This study has two major strengths. One that is a longitudinal study in comparison to one-time measures studies, which are lacking in the frailty literature, which allows for a better understanding of the adverse outcome prediction (Hwang et al., 2021). And second, albeit the retrospective nature of the study, the images utilized here were obtained prospectively, so imaging protocols, settings, and acquisition were standardized in the previous study, something that is not common in radiomic studies (Van Timmeren et al., 2020).

Our study must also be interpreted with caution as the findings are based on a limited sample size from a single center. As with other radiomics studies, there is an inherent need for a high number of patients, otherwise, it leads to overfitting of data or finding relationships where there are none, also called false discovery (Limkin et al., 2017). The limited number of adverse events in this study (i.e., only two patients develop hearing impairment over a median follow-up of 2 years) means that further testing in independent cohorts is needed to refine and calibrate the radiomic model. Confirmation of our results in studies with more patients and longer follow-up time is therefore warranted (Oikonomou et al., 2019). However, the best way to assess the potential clinical value of a model is validation with prospectively collected independent cohorts, ideally clinical trials (Rizzo et al., 2018). A future prospective cohort or analysis of retrospectively randomized clinical data is required to validate the radiomic phenotype (Lu et al., 2019).

Another limitation of this methodology is that unfortunately, neither the computational feature by traditional radiomics nor the use of machine learning or deep network learning can explain the function of radiomics for predicting clinical events. The interpretability of this method is not strong, and in particular, explanations of the underlying biological and molecular mechanisms are lacking to none existent (Gao et al., 2021). Although we have performed a robust statistical analysis, it should be emphasized that radiomic analyses can be used to identify correlations or associations, not causes; thus, they are not expected to enable definitive assessment of contents of tissue through imaging alone (Gillies et al., 2016). That is why one of the major drawbacks of this study is the lack of biological data for comparison and validation purposes. Efforts to introduce biological meaning into radiomics are gaining traction in the field with distinct approaches available (Tomaszewski and Gillies, 2021).

Furthermore, radiomics in general still faces many challenges, including the stability and reproducibility of the developed phenotypes or predictive models, as well as the interpretability before it can be translated to clinical applications (Ibrahim et al., 2021). To account for these sources of variability researchers in radiomics need to image at multiple time points, perform phantom studies, and analyzed how sensitive the radiomics models are for different segmentation methods. However, as with any emerging methodology, proof-of-concept investigations need to be first conducted to see what the potential applications and limitations of a new methodology are (Kolossvary and Maurovich-Horvat, 2019).

### Future Directions

It is hypothesized that phenotype similarities of different disorders may indicate biological relationships of the underlying genes (Scharfe et al., 2009). Mitochondrial dysfunction has been identified in cancer, infertility, diabetes, heart diseases, blindness, deafness, stroke, dwarfism, and resulting from numerous medication toxicities. Mitochondrial dysfunction is also involved in normal aging and age-related neurodegenerative diseases, such as Parkinson’s and Alzheimer’s diseases (Goldstein and Wolfe, 2013). The pathogenesis of muscle dysfunction in the aging individual is multifaceted and encompasses lifestyle habits, systemic factors, local environment perturbations, and intramuscular specific processes. However, in this scenario, derangements in skeletal myocyte mitochondrial function are also recognized as major factors contributing to the age-dependent muscle dysfunction (Adelnia et al., 2019; Liu et al., 2021). Although mitochondrial defects affect many cellular processes, the phenotype patterns predominantly represent deficiencies in energy metabolism Zampino et al., 2020 with the nervous system and the cardiac system being most susceptible. Thus, our results showing a higher proportion of neurological (e.g., dementia, stroke, and hearing impairment), cardiological (e.g., myocardial infarction), associated with muscle dysfunction (e.g., falls, ultrasonic muscle changes), lead to the proposed hypothesis that the muscle radiomic phenotype could be due to mitochondrial dysfunction.

In the future, we hope to connect our radiomics data with other data such as genetics, transcriptomics, proteomics, or metabolomics to achieve better identification of actual pathways of disease progression and to investigate evolving physiology in this field (Gillies et al., 2016). Data sets could be used as an initial framework for linking genes and pathways to clinical or radiological phenotypes (Calvo and Mootha, 2010). We believe that the mitochondrial theory of aging hypothesis could also be supported by image-guided biopsies or the use of circulating cell-free mitochondrial DNA, which could demonstrate that the imaging muscle shows spatial differences in protein expression, gene expression, etc. Some authors suggest that image-guided proteomics holds promise for characterizing tissues (Lambin et al., 2012) and pay the way for understanding the physiological and molecular mechanism of aging.

### Conclusion

Although this study has several limitations, including its retrospective nature, a small number of events and patients, and the lack of biological correlation, it demonstrates the feasibility and usefulness of radiomics methodology for building prognostic image-based phenotyping in the elderly and frail population. The muscle radiomic model needs to further be validated. Future studies correlated with biological data (genomics, transcriptomics, metabolomics, etc.) will give further insights into the biological basis and molecular processes of the developed radiomic model.

## Data Availability

The raw data supporting the conclusion of this article will be made available by the authors, without undue reservation.
