# Supplementary material for: The Usefulness of Radiomics Methodology for Developing Descriptive and Prognostic Image-Based Phenotyping in the Aging Population: Results From a Small Feasibility Study
Source: Front Aging. 2022 Apr 28;3:853671. doi: 10.3389/fragi.2022.853671 (PMC9261370; doi:10.3389/fragi.2022.853671)
Supplement: Supplementary file 1 [file DataSheet1.pdf]

1. Supplementary Material

1.1. Appendix 1. Assessment of Frailty Phenotype. For the assessment of frailty phenotype, the criteria proposed by Linda Fried and collaborators in 2001 were used (Fried et al., 2001). This includes unintentional weight loss, feeling of general exhaustion, low level of physical activity, slow walking speed, and muscular weakness. The methods used to evaluate each criterion are described here: (a) Unintentional weight loss: It was defined as an unintentional weight loss of 4.5 Kg or more during the past year. This information was obtained through the question, "In the last year, have you lost more than 4.5 Kg unintentionally (not due to diet or exercise)?" (b) Increased tiredness or exhaustion: This was self-reported by the subject, answering affirmatively to any of the following questions: "I feel that everything I did was a great effort in the last 3 or 4 days" and/or "In the last month, I had too little energy to do things I usually like to do". (c) Low level of physical activity: A positive response to either two statements: “I do not do physical activity” or “I do no more than one or two walks per week”. (d) Slow walking speed: Defined as a speed lower than predetermined values according to the sex and height of the subject in the speed test of the march at a distance of 4.6 meters, following the provisions of the "Short Physical Performance Battery" (Guralnik, Seeman, Tinetti, Nevitt, & Berkman, 1994). Aids like cane, walker, or crutches were allowed for walking. Since the goal was to determine their usual walking speed, subjects were not encouraged to go fast. To avoid the biases of the reaction time, the initial acceleration, and the possible final deceleration, both at the exit of the test and its arrival, the exit and arrival signals were located 0.50 m away from the start and the end of the footage tested (4.6 m). (e) Muscle weakness: The contraction force or muscle grip of the dominant hand of the patients was measured (Cooper et al., 2013; Cruz-Jentoft et al., 2010) in kilograms (Kg) with a hand-held digital dynamometer (Trailite Steiner, TL-LSC100 Coesfeld, Germany). The patients were seated with the elbow flexed at a right angle along the body, the wrist in the neutral position, and the hand on the handle of the dynamometer (Massy-Westropp, Gill, Taylor, Bohannon, & Hill, 2011). Each subject was asked to make three attempts with their maximum sustained effort for 15 seconds, with 30 seconds of recovery between each measurement. In cases in which the patient did not have dominant laterality, the measurement was made in the right hand. For data analysis, the average of the three attempts was used, and muscle weakness was defined as a value in Kgs below predetermined values, adjusted for sex and BMI (Kg/m^2). Even if lower limbs are more relevant for a physical function such as upper limbs, handgrip strength has been widely used and is well correlated with most relevant outcomes (Chan, van Houwelingen, Gussekloo, Blom, & den Elzen, Wendy P. J., 2014). A point was assigned to each positive criterion, constructing a final score formed by the sum of the five criteria. According to this score, subjects were classified as follows: (a) Robust: 0 points; (b) Prefrail: 1 or 2 points; and (c) Frail: 3 or more points.

1.2. Table 1S. List of Radiomic Features Used in the Image Analysis and Definition of Radiomic Features (Adapted from Vallières, et al. 2015). The texture of an image region is determined by the way the gray levels are distributed over the pixels in the region. The features describe and quantify the properties of an image region by exploiting space relations underlying the gray-level distribution of a given image. In first-order statistical texture analysis, texture information is extracted from the histogram of image intensity. This approach measures the frequency of a

particular grey level at a random image position and does not take into account correlation, or co-occurrences, between pixels. In second-order statistical texture analysis, texture information is based on the probability of finding a pair of grey levels at random distances and orientations over an entire image. We now list the texture features applied in this study.

Table 1S. List of Radiomic Features Used in the Image Analysis

Group	Number of features	*Name of the radiomic features and code used in the radiomic analysis*
Intensity histogram or first-order statistics	4	Mean (1)
		Variance (2)
		Skewness (3)
		Kurtosis (4)
Gray-level co-occurrence matrix (GLCM)	9	Energy (5)
		Contrast (6)
		Entropy (7)
		Homogeneity (8)
		Correlation (9)
		Sum Average (10)
		Variance (11)
		Dissimilarity (12)
		Auto-Correlation (13)
Gray-level run-length matrix (GLRLM)	13	Short Run Emphasis (14)
		Long Run Emphasis (15)
		Gray-Level Nonuniformity (16)
		Run Length Nonuniformity (17)
		Run Percentage (18)
		Low Gray-Level Run Emphasis (19)
		High Gray-Level Run Emphasis (20)
		Short Run Low Gray-Level (21)
		Short Run High Gray-Level (22)
		Long Run Low Gray-Level (23)
		Long Run High Gray-Level (24)
		Gray-Level Variance (25)
		Run-Length Variance (26)
Gray-level size zone matrix	13	Small Zone Emphasis (27)

(GLSZM)		Large Zone Emphasis (28)
		Gray-Level Nonuniformity (29)
		Zone-Size Nonuniformity (30)
		Zone Percentage (31)
		Low Gray-Level Zone Emphasis (32)
		High Gray-Level Zone Emphasis (33)
		Small Zone Low Gray-Level Zone Emphasis (34)
		Small Zone High Gray-Level Zone Emphasis (35)
		Large Zone Low Gray-Level Zone Emphasis (36)
		Large Zone High Gray-Level Zone Emphasis (37)
		Grey-Level Variance (38)
		Zone-Size Variance (39)
Neighborhood gray-tone difference matrix (NGTDM)	5	Coarseness (40)
		Contrast (41)
		Busyness (42)
		Complexity (43)
		Strength (44)

1.3. Table 2S. Baseline Characteristics of the Sample According to Frailty Phenotype

**Table 2S. Baseline Characteristics of the Sample According to Frailty Phenotype**						
Group	***Controls***	***Robust***	***Pre-frail***	***Frail***	***Total***	***Statistical Test***
	(n = 24)	***(n = 22)***	***(n = 30)***	***(n = 25)***	***(n = 101)***	
Variable	***Mean (SD)***	***Mean (SD)***	***Mean (SD)***	***Mean (SD)***	***Mean (SD)***	***P-value***
Physical Characteristics						
Age (years)	43(12)	68 (6)	73 (7)	74 (8)	65 (15)	<0,001*
Weight (Kg)	72,6 (13,5)	74,2 (11,0)	75,8 (15,5)	74,8 (15,5)	74,4 (14,0)	0,826*
Height (m)	1,67 (0,08)	1,66 (0,08)	1,64 (0,09)	1,58 (0,09)	1,64 (0,09)	0,012
BMI (kg/m2)	26,1 (3,8)	26,8 (3,5)	28,1 (4,2)	29,8 (5,7)	27,8 (4,6)	0,022
Gait Speed (s)	2,7 (0,6)	2,9 (0,5)	3,7 (0,7)	5,0 (1,2)	3,6 (1,2)	<0,001*
MS (Kg)	30,49 (12,5)	29,12 (13,8)	25,24 (10,5)	23,31 (9,2)	26,86 (11,7)	0,204*
Ultrasound Measurements						
SFT (cm)	1,2 (0,5)	0,9 (0,5)	1,1 (0,5)	1,1 (0,7)	1,1 (0,6)	0,372*
MT (cm)	3,25 (0,59)	2,44 (0,54)	2,41 (0,64)	2,08 (0,54)	2,53 (0,71)	<0,001
Quality of Life (A.U.)	119 (5,4)	122 (6,6)	120 (4,8)	115 (7,6)	119 (6,6)	0,001

Sex	*N (%)*	*N (%)*	*N (%)*	*N (%)*	*N (%)*	0,235*
Female	11 (45,8)	6 (27,3)	15 (50)	14 (56)	46 (45,5)	
Male	13 (54,2)	16 (72,7)	15 (50)	11 (44)	55 (54,4)	
Frailty Criteria						<0,001*
No positive criterion	17 (70,1)	22 (100)	0	0	39 (38,6)	
1 positive criterion	5 (20,8)	0	13 (43,3)	0	18 (17,8)	
2 positive criteria	2 (8,3)	0	17 (56,6)	0	19 (18,8)	
3 positive criteria	0	0	0	11(44)	11 (10,9)	
4 positive criteria	0	0	0	7 (28)	7 (6,9)	
5 positive criteria	0	0	0	7 (28)	7 (6,9)	
**Abbreviations: SD: standard deviation; BMI: body mass index; MS: muscle strength; MT: muscle thickness; SFT: subcutaneous fat thickness; AU: arbitrary units.**						
* Variables without normal distribution, nonparametric tests used for the analysis.						

1.4. Table 3S. Comorbidities at Baseline and Two-Year Follow-up According to Frailty Phenotype

Table 3S. Comorbidities at Baseline and at Two-Year Follow-up According to Frailty Phenotype

Time Frame	***Baseline (at time of ultrasound scanning)***						***After 2 years of follow-up***					
Group	***Controls***	***Robust***	***Pre-frail***	***Frail***	***Total***	***Statistical Test***	***Controls***	***Robust***	***Pre-frail***	***Frail***	***Total***	***Statistical Test***
	(n = 24)	***(n = 22)***	***(n = 30)***	***(n =25)***	***(n=101)***		***(n = 24)***	***(n = 22)***	***(n = 30)***	***(n =25)***	***(n=101)***	
Variable	***N (%)***	***N (%)***	***N (%)***	***N (%)***	***N (%)***	***P***	***N (%)***	***N (%)***	***N (%)***	***N (%)***	***N (%)***	***P***
Associated Diseases												
Hypertension	4 (16,6)	8 (36,3)	13 (43,3)	15 (60)	40 (39,6)	0,020*	7 (29,2)	9 (40,9)	20 (66,7)	20 (80)	56 (55,4)	0,001*
Hyperlipidemia	9 (37,5)	10 (45,4)	22 (73,3)	16 (64)	57 (56,4)	0,036*	10 (41,6)	15 (68,1)	22 (73,3)	20 (80)	67 (66,3)	0,026*
DM	0	6 (27,3)	10 (33)	10 (40)	26 (25,7)	0,008*	1 (4,2)	7 (31,8)	12 (40)	10 (48)	30 (29,7)	0,017*
COPD	0	2 (9)	5 (16,7)	5 (20)	12 (11,8)	0,134*	0	2 (9)	5 (16,7)	7 (28)	14 (13,8)	0,035*
Hearing Impairment	3 (12,5)	4 (18,2)	7 (23,3)	6 (24)	20 (19,8)	0,719*	3 (12,5)	4 (18,1)	8 (26,7)	7 (28)	22 (21,7)	0,504*
Visual Impairment	5 (20,8)	11 (50)	14 (46,7)	15 (60)	45 (44,5)	0,044*	3 (12,5)	14 (63,6)	17 (56,7)	16 (64)	50 (49,5)	0,001*
Parkinson Disease	0	0	2 (6,7)	0	2 (1,9)	0,189*	0	0	2 (6,7)	1 (4)	3 (2,9)	0,408*
Previous Stroke	0	0	2 (6,7)	3 (12)	5 (4,9)	0,160*	0	0	2 (6,7)	7 (28)	9 (8,9)	0,001*
Congestive Heart Failure	1 (4,2)	1 (4,5)	0	3 (12)	5 (4,9)	0,241*	1 (4,2)	1 (4,5)	0	5 (20)	7 (6,9)	0,026*
Heart Disease	3 (12,5)	3 (13,6)	5 (16,7)	7 (28)	18 (17,8)	0,476*	3 (12,5)	5 (22,7)	8 (26,7)	12 (48)	28 (27,7)	0,044*
Myocardial Infarction	1 (4,2)	1 (4,5)	0	0	2 (1,9)	0,489*	1 (4,2)	1 (4,5)	2 (6,7)	5 (20)	9 (8,9)	0,164*
Renal Disease	1 (4,2)	2 (9)	5 (16,7)	9 (36)	17 (16,8)	0,017*	1 (4,2)	7 (31,8)	9 (40,9)	12 (48)	29 (28,7)	0,009*
Previous Cancer	0	5 (22,7)	7 (23,3)	5 (20)	17 (16,8)	0,093*	0	5 (22,7)	6 (20)	8 (32)	19 (18,8)	0,036*

Arthritis / Osteoarthritis	2 (1,9)	12 (54,5)	21 (70)	16 (64)	51 (50,4)	<0,001*	3 (12,5)	13 (59)	24 (80)	17 (68)	57 (56,4)	<0,001*
Anxiety / Depression	3 (12,5)	7 (31,8)	10 (33,3)	15 (60)	35 (34,6)	0,007*	4 (16,7)	7 (31,8)	12 (40)	16 (64)	39 (38,6)	0,007*
Previous fractures / Osteoporosis	1 (4,2)	3 (13,6)	7 (23,3)	8 (32)	19 (18,8)	0,074*	2 (8,3)	5 (22,7)	10 (33,3)	9 (36)	26 (25,7)	0,106*
Liver Disease / Hepatopathy	1 (4,2)	0	1 (3,3)	5 (20)	7 (6,9)	0,029*	1 (4,2)	0	2 (6,7)	5 (20)	8 (7,9)	0,062*
Dementia / Memory Loss	0	0	0	1 (4)	1 (0,9)	0,385*	0	2 (9,1)	5 (16,7)	5 (20)	12 (11,8)	0,134*
Connective Tissue Disease	0	0	0	0	0	1,000*	1 (4,2)	2 (9,1)	0	0	3 (2,9)	0,202*
Hemiplegia	0	0	0	0	0	1,000*	0	0	0	1 (4)	1 (0,9)	0,385*
Neoplasm	0	0	0	0	0	0,385*	1 (4,2)	2 (9,1)	4 (13,3)	3 (12)	10 (9,9)	0,704*
Leukemia/Malignant Lymphoma	0	0	0	0	0	1,000*	0	0	1 (3,3)	1 (4)	2 (1,9)	0,632*
Solid Metastasis	0	0	0	0	0	1,000*	0	1 (4,5)	0	0	1 (0,9)	0,309*
AIDS	0	0	0	0	0	1,000*	0	0	0	0	0	1,000*
Peripheral Vascular Disease	0	0	1 (3,3)	1 (4)	2 (1,9)	0,632*	0	1 (4,5)	0	2 (8)	3 (2,9)	0,262*
Risk Factors												
Smoker	8 (33,3)	4 (18,2)	10 (33)	8 (32)	30 (29,7)	0,618*	8 (33,3)	4 (18,1)	9 (30)	8 (32)	29 (28,7)	0,663*
Alcohol	2 (8,3)	1 (4,5)	0	2 (8)	5 (4,9)	0,454*	2 (8,3)	1 (4,5)	0	2 (8)	5 (4,9)	0,454*
Falls	0	0	3 (10)	3 (12)	6 (5,9)	0,146*	0	0	6 (20)	5 (20)	11 (10,8)	0,017*
Obesity	0	2 (9)	1 (3,3)	6 (24)	9 (8,9)	0,015*	1 (4,2)	2 (9,1)	2 (6,7)	7 (28)	12 (11,8)	0,038*
Associated with Frailty												
# of visits to PC	4 (16,7)	5 (22,7)	5 (16,7)	7 (28)	5 (4,9)	0,077*	4 (16,7)	5 (22,7)	6 (20)	9 (45)	6 (5,6)	0,001*
# of visits to ED	1 (4,2)	1 (4,5)	1 (3,3)	1 (4)	1 (0,9)	0,177*	1 (4,2)	1 (4,5)	1 (3,3)	2 (8)	1 (0,9)	0,001*
# of Hospitalizations	0	0	0	1 (4)	1 (0,9)	0,243*	0	0	0	1 (4)	1 (0,9)	0,085*

Abbreviations: N: number of positive cases; SD: standard deviation; DM: diabetes mellitus; COPD: chronic obstructive pulmonary disease; AIDS: acquired immune deficiency syndrome; PC: primary care; ED: emergency department, #: number.

* Variables without normal distribution, nonparametric tests used for the analysis.

1.5. Table 4S. List of Radiomic Features Used in the Radiomic Phenotype Models

Table 4S. List of Radiomic Features Used in the Radiomic Phenotype Models		
Radiomic model and number of features	***Hierarchical Order***	***Name of the radiomic variables and corresponding code in the radiomic analysis***
Radiomic Phenotype Model 1 (26 radiomic fetures)	44	Small Zone Emphasis of Gray-level size zone matrix (27)
	43	Zone-Size Nonuniformity of Gray-level size zone matrix (30)
	42	Complexity of Neighborhood gray-tone difference matrix (43)
	41	Short Run Emphasis of Gray-level run-length matrix (14)
	40	Run Length Nonuniformity of Gray-level run-length matrix (17)
	39	Run Percentage of Gray-level run-length matrix (18)

	38	Zone Percentage of Gray-level size zone matrix (31)
	37	Entropy of Gray-level co-occurrence matrix (7)
	36	Variance of Gray-level co-occurrence matrix (11)
	35	Variance of pixels (2)
	34	Gray-Level Variance of Gray-level run-length matrix (25)
	33	Contrast of Gray-level co-occurrence matrix (6)
	32	Dissimilarity of Gray-level co-occurrence matrix (12)
	31	Contrast of Neighborhood gray-tone difference matrix (41)
	30	Grey-Level Variance of Gray-level size zone matrix (38)
	29	Strength of Neighborhood gray-tone difference matrix (44)
	28	Correlation of Gray-level co-occurrence matrix (9)
	27	Auto-Correlation of Gray-level co-occurrence matrix (13)
	26	High Gray-Level Run Emphasis of Gray-level run-length matrix (20)
	25	Short Run High Gray-Level of Gray-level run-length matrix (22)
	24	High Gray-Level Zone Emphasis of Gray-level size zone matrix (33)
	23	Small Zone High Gray-Level Zone Emphasis of Gray-level size zone matrix (35)
	22	Sum Average of Gray-level co-occurrence matrix (10)
	21	Long Run High Gray-Level of Gray-level run-length matrix (24)
	20	Large Zone High Gray-Level Zone Emphasis of Gray-level size zone matrix (37)
	19	Mean of pixel (1)
Radiomic Phenotype Model 2 (4 radiomic features)	18	Large Zone Low Gray-Level Zone Emphasis of Gray-level size zone matrix (36)
	17	Coarseness of Neighborhood gray-tone difference matrix (40)
	16	Long Run Low Gray-Level of Gray-level run-length matrix (23)
	15	Run-Length Variance of Gray-level run-length matrix (26)
Radiomic Phenotype Model 3 (14 radiomic features)	14	Gray-Level Nonuniformity of Gray-level run-length matrix (16)
	13	Gray-Level Nonuniformity of Gray-level size zone matrix (29)
	12	Energy of Gray-level co-occurrence matrix (5)
	11	Low Gray-Level Zone Emphasis of Gray-level size zone matrix (32)
	10	Small Zone Low Gray-Level Zone Emphasis of Gray-level size zone matrix (34)
	9	Low Gray-Level Run Emphasis of Gray-level run-length matrix (19)
	8	Short Run Low Gray-Level of Gray-level run-length matrix (21)
	7	Kurtosis of pixels (4)
	6	Long Run Emphasis of Gray-level run-length matrix (15)
	5	Large Zone Emphasis of Gray-level size zone matrix (28)
	4	Homogeneity of Gray-level co-occurrence matrix (8)
	3	Zone-Size Variance of Gray-level size zone matrix (39)
	2	Busyness of Neighborhood gray-tone difference matrix (42)
	1	Skewness of pixels (3)

1.6. Table 5S. Characteristics of the Sample According to Identified Radiomic Phenotype

**Table 4S. Characteristics of the Sample According to Identified Radiomic Phenotype**				
Group	***No*** (*n = 79*)	***Yes*** (*n = 22)*	***Total*** (*n = 101)*	***Statistical Test***
Variable	***Mean (SD)***	***Mean (SD)***	***Mean (SD)***	***P-value***
Physical Characteristics				
Age (years)	62 (15)	75 (6)	65 (15)	<0,001*
Weight (Kg)	75,0 (13,5)	72,3 (15,7)	74,4 (14,0)	0,428*
Height (m)	1,64 (0,08)	1,62 (0,09)	1,64 (0,09)	0,288
BMI (kg/m2)	27,8 (4,5)	27,6 (4,8)	27,8 (4,6)	0,822
Gait Speed (s)	3,4 (1,1)	4,4 (1,3)	3,6 (1,2)	<0,001*
MS (Kg)	27,57 (11,8)	24,29 (10,7)	26,86 (11,7)	0,246*
Ultrasound Measurements				
SFT (cm)	1,1 (0,6)	0,9 (0,5)	1,1 (0,6)	0,033*
MT (cm)	2,65 (0,7)	2,11 (0,8)	2,53 (0,71)	0,001
Quality of Life (A.U.)	119 (6,4)	116 (6,4)	119 (6,6)	0,013*
Sex	***N (%)***	***N (%)***	***N (%)***	0,992
Female	36 (78,3)	10 (21,7)	46 (45,5)	
Male	43 (78,1)	12 (21,9)	55 (54,4)	
Frailty Phenotype				<0,001*
Control	24 (100)	0 (0)	24 (23,8)	
Robust	20 (90)	2 (10)	22 (21,7)	
Pre-Frail	21 (70)	9 (30)	30 (29,7)	
Frail	14 (56)	11 (44)	25 (24,8)	

Abbreviations: SD: standard deviation; BMI: body mass index; MS: muscle strength; MT: muscle thickness; SFT: subcutaneous fat thickness; AU: arbitrary units.

* Variables without normal distribution, nonparametric tests used for the analysis.

1.7. **Figure 1S. (A) Heatmap of Correlation coefficients for an association of Radiomic Features and Epidemiological Data (n = 101).** On the x-axis, radiomics features are shown, and on the y-axis are the physical characteristics, ultrasound measurements, frailty criteria, and quality of life. The elements of the heatmap are color-coded depending on the value of the correlation coefficient. Red is for the highest value and green for the lowest, with 5 different colors in between. **Abbreviations:** BMI: body mass index; MS: muscle strength; MT: muscle thickness; SFT: subcutaneous fat thickness; OPQoL: Older´s People Quality of Life; AU: arbitrary units; IHF: Intensity Histogram Features; GLCM: Gray-Level Co-occurrence Matrix; GLRLM: Gray-Level run-Length Matrix; GLSZM: Gray-Level Size Zone Matrix; NGTDM: Neighborhood Gray-Tone Difference Matrix. **Note:** Rho of Spearman was used for the statistical analysis. **(B) Manhattan plot of p-values for associations between radiomic features and epidemiological data.** P-values for univariate associations between each radiomic feature and physical characteristics (age, weight, height, BMI), muscle performance data (muscle strength, gait speed, and anterior thigh subcutaneous fat tissue and muscle thickness), frailty phenotype, and quality of life taken during the baseline ultrasound. Radiomic features are situated on the x-axis in the same order as the heatmap, while the corresponding p-values are located on the y-axis and graph

with a -LOG10(p-value) scale. Points above the red line (p= <0.05) indicate radiomic features showed significant association with the above-named variables.

(A)

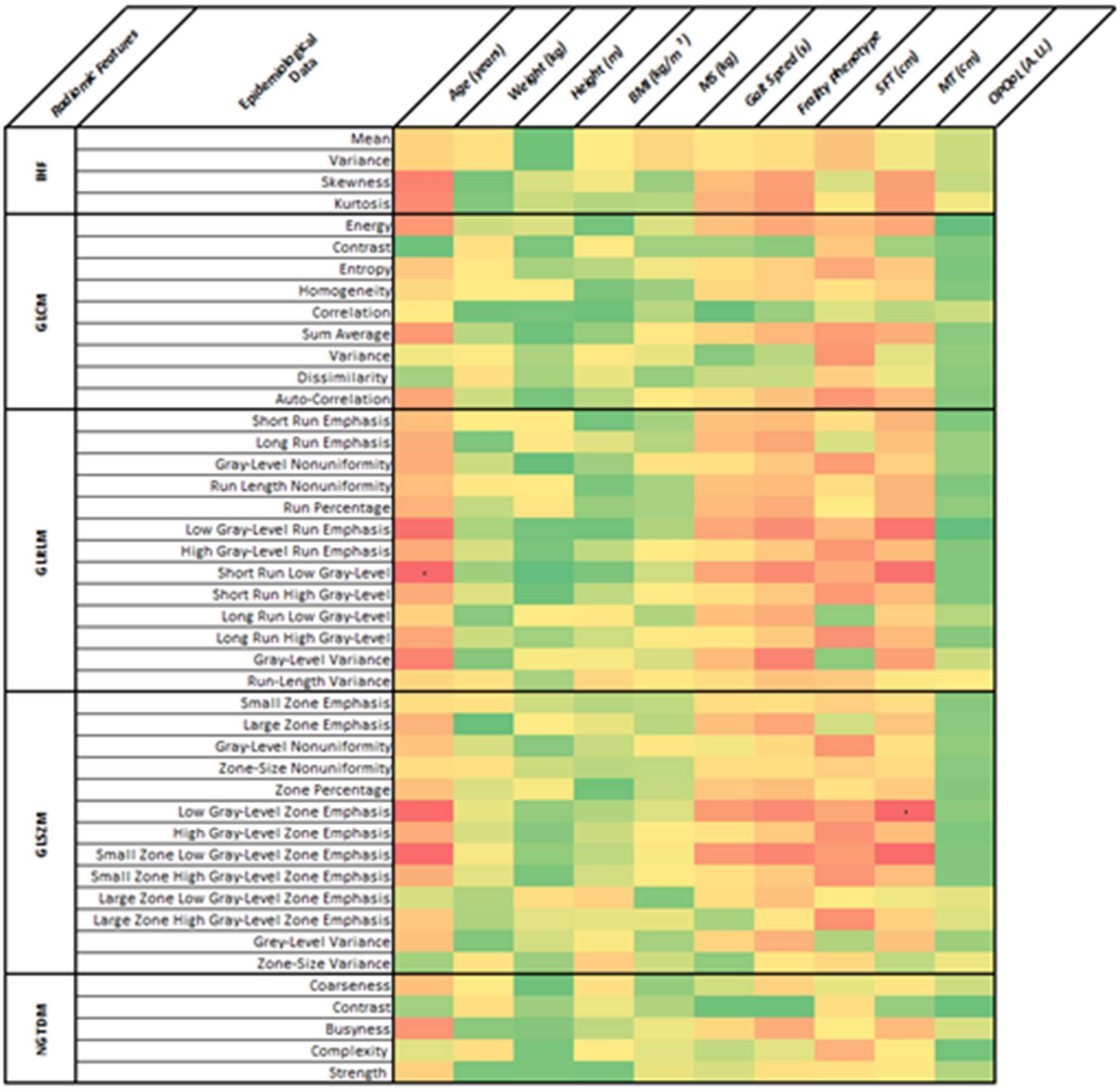

(B)

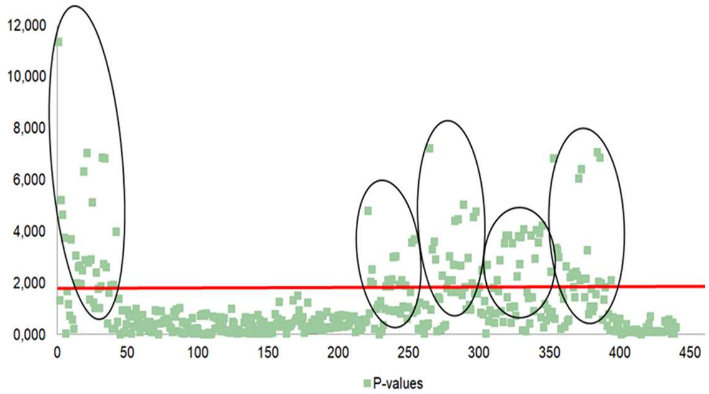
12,000
10,000
8,000
6,000
4,000
2,000
0,000
0
50
100
150
200
250
300
350
400
450
P-values
